# Linking Social and Vocal Brains: Could Social Segregation Prevent a Proper Development of a Central Auditory Area in a Female Songbird?

**DOI:** 10.1371/journal.pone.0002194

**Published:** 2008-05-21

**Authors:** Hugo Cousillas, Isabelle George, Laurence Henry, Jean-Pierre Richard, Martine Hausberger

**Affiliations:** Université de Rennes 1, Ethos, UMR 6552 CNRS – Ethologie animale et humaine, Rennes, France; National Institutes of Health, United States of America

## Abstract

Direct social contact and social interaction affect speech development in human infants and are required in order to maintain perceptual abilities; however the processes involved are still poorly known. In the present study, we tested the hypothesis that social segregation during development would prevent the proper development of a central auditory area, using a “classical” animal model of vocal development, a songbird. Based on our knowledge of European starling, we raised young female starlings with peers and only adult male tutors. This ensured that female would show neither social bond with nor vocal copying from males. Electrophysiological recordings performed when these females were adult revealed perceptual abnormalities: they presented a larger auditory area, a lower proportion of specialized neurons and a larger proportion of generalist sites than wild-caught females, whereas these characteristics were similar to those observed in socially deprived (physically separated) females. These results confirmed and added to earlier results for males, suggesting that the degree of perceptual deficiency reflects the degree of social separation. To our knowledge, this report constitutes the first evidence that social segregation can, as much as physical separation, alter the development of a central auditory area.

## Introduction

Over the last decade the importance of social influences on vocal development has become an evidence in a variety of species [1], [2]. Recent studies reveal how social cues affect speech development in human infants [3], and also how direct social contacts and interactions are required for infants to maintain perceptual abilities to discriminate phonetic units [4]. Attention and motivation are key elements in learning to communicate: children involved in a social situation are more “awake” and attentive, and therefore more prone to react and memorize [5]. Thus, early awareness of infant is a good predictor of their later language skills [6]. Social interactions activate attentional processes, enabling the processing and integration of information [7], while the intersensory redundancy they provide facilitates attentional focusing on certain aspects of the sensory stimulation [8]. However, the processes involved in this link between “language brain” and “social brain” are still poorly known: the interface between language and social cognition remains a mystery [2].

Songbirds are good candidates for trying to unravel this mystery: like humans, they are sensitive to social influences for vocal learning and they are active in their choice of tutor [e.g. 9]. Again, the exact processes involved are not well known, but here also social stimulations may enhance attention and arousal, as well as motivation. According to Hultsch et al. [10], the positive effects of social exposure on song learning could come from perceptual mechanisms that make young birds more attentive to the tutors' vocalizations. Indeed, socially deprived birds appear to show hearing deficits [11]. Interestingly, visual stimuli may activate auditory central parts of a songbird's brain [12]. Moreover, selective attention is one of the processes that may alter hearing by changing the micromechanical properties of the cochlea [13]. This could explain that vocal copying, as well as perceptual abilities, are tuned on particular tutors [e.g. 4]. Social bonding appears essential in many social songbird species, as well as in humans, for vocal learning [14], and one wonders what consequences the lack of such a bond would have. Children that interact more with peers than with adults develop poorer language skills [15], and neglected children show poorer language abilities than normally developing children, but also than abused children [16]. The fact that autistic children, who are characterized by impairments of their social interactions, also present selective impairments in attention to vocal-speech sounds [17], and abnormal cortical voice processing [18], further emphasizes the link between social and perceptual development.

In this present pioneering study, we aim to improve our knowledge of this link by testing the hypothesis that social segregation prevents the proper development of a central auditory area. Our previous studies showed that neuronal preferences and general characteristics of development (proportion of auditory sites, response types) of the field L (which is a homologue of the primary auditory cortex of mammals) of male European starlings depend not only on early auditory experience during development [19], but also on social experience per se. Thus, young male starlings that could hear adult song, but were socially deprived, showed, when adult, deficits similar to those of auditory deprived animals: larger auditory area, poor selectivity, altered tonotopy [20], recalling findings for auditory deprived young rats [21]. Still more intriguing was the finding that young males raised in direct contact with adult males, although presenting a much better structured auditory area than the above-mentioned deprived animals, also showed consistent differences compared to wild-caught males, with a higher proportion of auditory sites and lower neuronal specialization. As these young males preferentially developed bonds with their peers, this suggested that social segregation from the adults, by lowering their selective attention towards their song, may have induced these abnormalities [22]. Social segregation has also been suggested to be responsible for limited recoveries in early deprived animals, when later they were placed with adults [23].

In order to test this hypothesis, we needed a situation where social segregation would be more clear-cut than in the previous study with young males. Therefore, we focused here on young females raised with male tutors. Female starlings are known to form strong same-sex social pairs, to prefer to sing near another female, and to learn song from same-sex tutors [22], [24], [25]. The aim was not to examine whether the effects would differ according to the tutor's sex, but to ensure that placing young females with male tutors would induce social segregation [26]; this has been confirmed by behavioural observations and song recordings [22]. Electrophysiological recordings performed on these females when they were adult revealed perceptual abnormalities that made these male-tutored females resemble more socially isolated birds than normal adult females. These findings agree with preliminary data for males, and constitute, to our knowledge, the first evidence that social segregation can, as much as physical separation [20], alter the development of a central auditory area.

## Results

**Figure 1:**
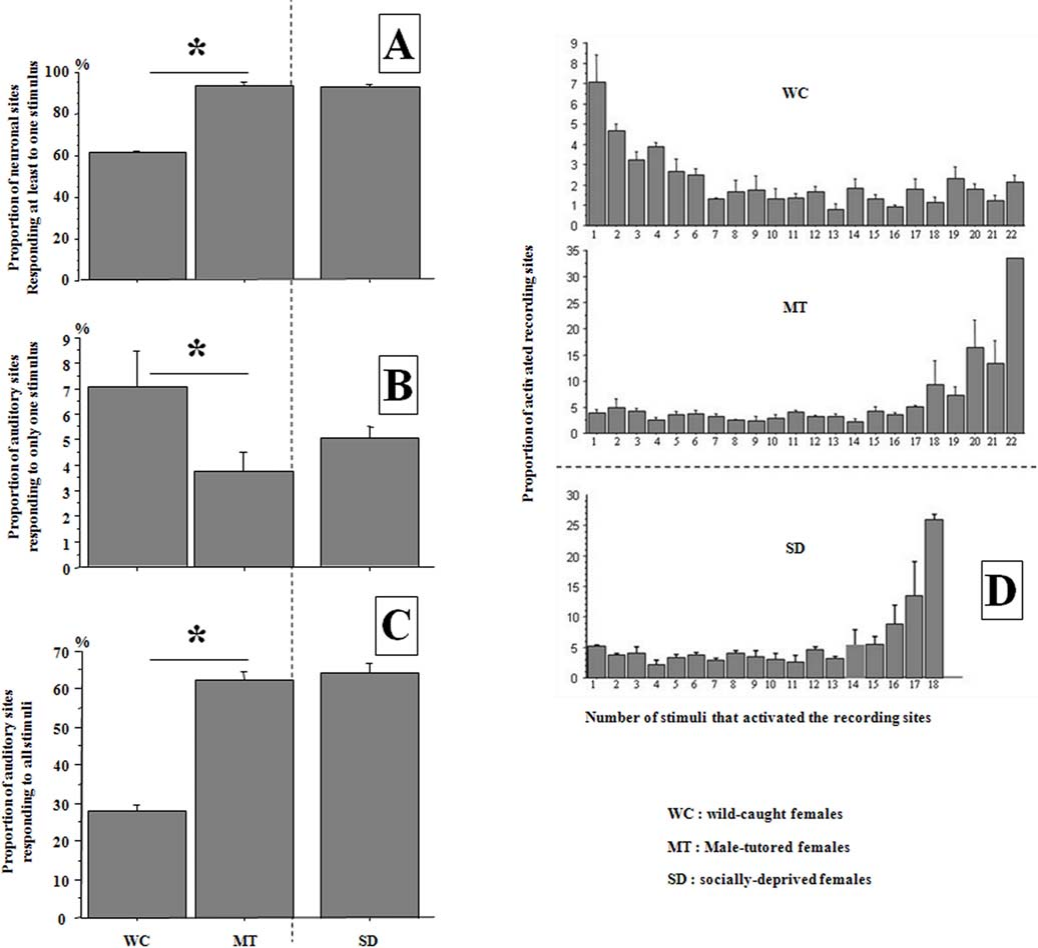
- A - Proportion of neuronal sites responding to at least one stimulus. The difference between wild-caught females (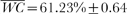) and male-tutored females (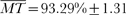) was significant (Mann-Whitney, U = 0, p = 0.05), but the proportion did not differ significantly between male-tutored and socially-deprived females (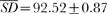). - B & C - Proportion of sites that responded to only one stimulus (B) and to all stimuli (C). These values were obtained using 6 class I whistles that were common to all birds. The difference between wild-caught and male-tutored females was significant in both cases (B- 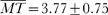, 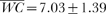; Mann-Whitney, U = 0, p = 0.05 and C- 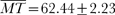, 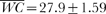, U = 0, p = 0.05). Results for male-tutored females and for socially deprived birds did not differ significantly. - D - Proportion of neuronal sites responding to the 1, 2..or all stimuli used. A majority of neuronal sites responded to 1 to 4 stimuli in the wild-caught females (WC), whereas most neuronal sites responded to all or most stimuli in the male-tutored (MT) as well as in the socially-deprived birds (SD).

We investigated the effects of adult male tutoring on the development of auditory responses of six hand-raised female European starlings (MT) when they had become adult (2 years old). We compared these results to those obtained for four adult wild-caught females (WC), by the same electrophysiological procedure. The use of the same procedure for every bird, based on systematic regular recordings in the same sagittal plane (2761 neuronal sites tested; 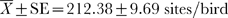; see material & method), enabled us to compare the number of responsive sites. This revealed clear differences between groups of birds. Indeed, the proportions of auditory sites significantly differed between the two groups (fig. 1A; 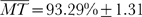, 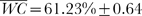; Mann-Whitney, U = 0, p = 0.05); the MT females showed a much higher proportion of responding sites than the WC females. We compared these data to an additional group of three females raised in social deprivation (SD, in pairs with one young male or isolated; see material & method), but that had heard the aviary vocal interactions through loudspeakers [see 22 for details]. Interestingly, the proportion of responsive sites of the MT birds was similar to that of the SD females (fig. 1A; 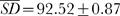). The MT females therefore appeared to be closer to deprived animals than to adult wild-caught birds. Note that their male peers were less affected as the proportions of their auditory sites differed significantly from that of SD animals (92%/98.5%), and male tutors (80%) [20]. These results clearly reflect the degree of social segregation and vocal copying, as young males, although staying mainly in same-age/same-sex groups, remained more in proximity and copied more their tutors than did the young females [22].

Neuronal specialization also differed between the two groups of females: a majority of neuronal sites in WC females responded to 1 to 4 stimuli, whereas most neuronal sites in the MT birds responded to all, or most stimuli, who again showed a pattern that was closer to that of SD animals (fig. 1D).

The proportions of specialized neurons were estimated by counting the recording sites that responded to 100% of the stimuli. This method gives a good indication of the number of non-specialized (or generalist) neurons in field L complex [19], [20]. As the fact that some types of stimuli (individual-specific whistle themes) were not common to all subjects could bias this evaluation, we compared here the responses to the six test stimuli that were common to all subjects (class I whistles). This analysis confirmed the preceding results: more auditory sites responded to only one stimulus in WC females than in MT females (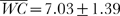; 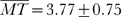; Mann-Whitney, U = 0, p = 0.05; fig. 1B), whereas generalist sites (responses to all stimuli) were clearly more numerous in MT females than in WC females (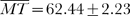, 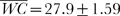, U = 0, p = 0.05 fig. 1C). Again, results for MT females were similar to those for SD animals (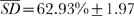). Note that, our results for MT males raised under the same conditions were intermediate: they presented a lower proportion of generalist neuronal sites than did SD animals (37%/46%), but a higher proportion than male tutors (2%) [20].

**Figure 2:**
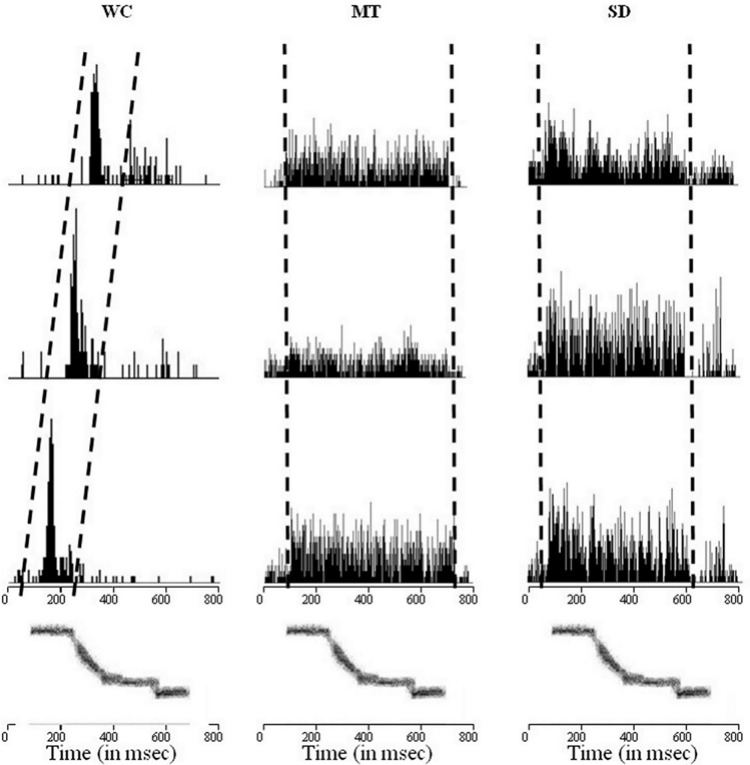
Examples of PSTHs calculated from three consecutive recording sites along a single penetration in a wild-caught (WC), a male-tutored (MT) and a socially-deprived (SD) Female. This example shows a pattern of selective responses at precise parameters typical of wild-caught birds: neurons responded to a restricted range of frequencies, shifting as frequency changed. While in male-tutored and socially-deprived birds, neurons were activated during the entire duration of the stimulus. The sonogram of the whistle used as stimulus is shown below the PSTHs.

Finally, PSTHs greatly differed between the two categories of animals subjects (fig. 2): as WC females showed a typical pattern of phasic selective responses to precise parameters of the stimuli, whereas MT animals showed a tonic, non-selective pattern. Again characteristics of MT females appeared close to those of SD animals.

## Discussion

Young female starlings raised with only peers and adult male tutors neither established close social bonds with males, nor did they copy their songs, restricting song sharing to peers that were equally inexperienced birds [22]. When tested as adults, it appeared that these females showed abnormalities in neuronal responses to the playback of species-specific stimuli in the main central auditory area (Field L), compared to WC adult females.

Several features were affected: they had 1) a larger auditory area (larger proportion of responsive sites), 2) a lower proportion of specialized neurons (sites responding to only one stimulus) and 3) a larger proportion of generalist sites (sites responding to all stimuli), associated with tonic, non-selective responses. Comparison with available data on SD birds showed similar abnormalities, suggesting that social segregation from adults may induce the same effects on perceptual development as a physical separation. These results are consistent with previous data on male peers who were, however, “intermediate” in that they differed not only from WC adults but also from SD young males. This reflected an intermediate social situation where young males, although forming mostly a same-sex/same-age group, showed some proximity with the adult males and copied some of their songs [20], [22].

These findings therefore strongly suggest that the degree of deficiency reflects the degree of social separation, be it physical or merely social segregation.

Overall, the observed abnormalities were similar to those described for other acoustically-deprived animals. Larger auditory areas have been observed in rats [21] and starlings [19] raised without proper auditory stimulation. Young male starlings deprived of auditory experience with adult song, also showed, when adult, a higher proportion of generalist, and lower proportion of specialized neuronal sites [19]. Interestingly, similar impairments were observed in birds that could hear adult song but had no contact with adults [20].

One could argue that the acoustic environment in the laboratory did not offer the variety of sounds that WC animals may experience in the field. However, first, the aviaries were placed in rooms with large windows allowing birds to hear sounds from outdoors (birds, dogs, cars etc., the usual sounds of a university campus) as well as from indoors, such as human voices, doors, other bird species, indicating that their acoustic environment was not totally impoverished. Second, while the acoustic environment could explain to some extent the differences observed between WC and MT females, it cannot explain the differences observed in males. Moreover, further experiments have shown that, under the same conditions, young males and females can develop normal song repertoires if they are placed in a dyadic situation with an adult, that is forced bonding, and do not if social bonding does not occur [26]. Finally, we have observed that young females do not learn better from playbacks of female songs than from playbacks of male songs, showing that mere auditory cues are very unlikely to be involved in sexual lines of learning [27]. Therefore, although acoustic conditions could explain some part of the differences observed between experimental and WC birds, it cannot fully explain it, which leaves room for the impact of social influence.

Central deficiencies in the auditory area clearly reflected differences in vocal copying according to social experience, both in the females that are described here and in the males that were raised with them. Thus, the fact that young males raised with an adult male did not copy much of the latter's song [20], [22] suggested that social segregation may have altered selective attention towards the tutor. The present results for females, which are even less prone to copy from adult males than young males, further reinforce this hypothesis. Since social influences may be mediated by attentional processes [22], [28], [29], the processing and integration of sensory information may have been altered [5], [7]. Selective attention has been shown to alter hearing by changing the micromechanical properties of the cochlea [13]. Moreover, Sturdy et al. [11] showed that zebra finches require social interactions with conspecifics to develop normal auditory perceptual abilities. Finally, Humans who lack experience with a language during development are considered “deaf” to some non-native language characteristics [30], and this is confirmed by Kuhl et al.'s [4] recent findings that infants need direct social interactions to maintain discriminative abilities.

The aim of the present study was to investigate in more depth the effects of social segregation on neural development suggested by previous studies [20], [23]. For that, we studied an extreme situation that we knew would not allow social bonding, even between birds in the same aviary, that is young females raised with male tutors [22], [24]. This extreme situation yielded the expected results, and confirmed preliminary findings for males. As we do not yet have data for females raised with adult females, no comparisons can be made. However, we expect that this situation would yield more “mixed” results, like those obtained for young males [26].

This pioneering study shows that young birds, when socially segregated from adults, exhibit abnormalities in the development of their central auditory area, and this to the same extent as socially-deprived animals. This confirmed previous indications in this direction. Mere environmental acoustic conditions cannot explain the entire array of evidence that this study, added to earlier reports [20], [23], has us enabled to present. The present results certainly add to the evidence that social and vocal brains are linked [2] and they shed new light on findings such as those of Gervais et al. [18] showing that socially-impaired autistic children present abnormal cortical voice processing. Indeed, the lack of social bonding due to the autistic syndrome might be responsible, through a lack of selective attention, for the perceptual impairments.

Further studies will be necessary to confirm our results that are, to our knowledge, the first ones to suggest an impact of “social isolation” on sensory development, and they have important general implications that go far beyond birdsong research.

## Materials and Methods

### Experimental animals

The experiment included two series of animals. (1) A “core” experiment with two groups of birds: one group of four wild-caught (WC) female starlings and one experimental group of six aviary-raised female starlings. The WC females were our “controls” as, in their wild environment; they had benefited from both female and male influences and were likely to have been able to learn their song from adult females [24]. The experimental females were raised in aviaries with peers (males and females) and only male adults [see 22]. The aviaries were in a room where all laboratory noises as well as external sounds (human voices, street traffic …) could be heard. (2) For a larger comparison we used additional data from three socially-deprived birds: one female raised in a pair with a male and two females raised in isolation in sound-proof chambers [22]. As no differences were evidenced between these two groups (e.g. mean proportions of responsive sites: raised in isolation = 92.10±1.32 and pair raised = 93.37), data from these three birds were pooled (socially deprived birds: SD). Note that, these birds could hear, through loudspeakers, the vocalizations emitted in the aviaries.

Data for song production of the experimental birds have been described in Poirier et al. [22] and revealed that the male-tutored (MT) females copied mainly songs of same-sex peers and very little songs of adult males.

### Stimuli

**Figure 3:**
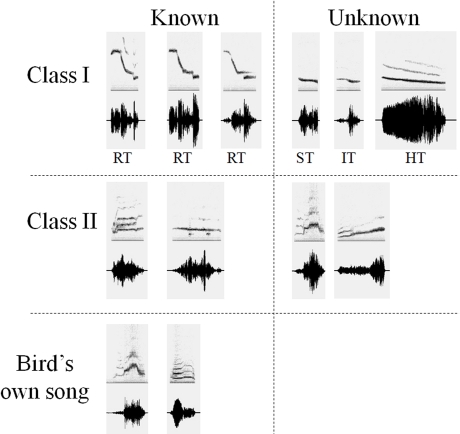
Examples of stimuli used in the experiment [20]. Every stimulus set included class-I universal whistles (RT: rhythmic theme's introductory notes - 3 examples from the 3 adult male tutors; ST: simple theme; IT inflection theme; HT: harmonic theme), and class-II individual whistles (whistles sung by familiar and unfamiliar males, and whistles from the bird's own repertoire). While all birds had the same class-I whistles, class II whistles differed among birds.

When the animals were 2 years old, neuronal responses to 22 species-specific stimuli were electrophysiologically tested, while the birds were awake and restrained, (fig. 3). The song repertoire of each female was recorded by placing them in individual sound-proof chambers and automatic song recordings were made until the complete repertoire of each bird was recorded [22]. The auditory stimuli were a variety of species-specific songs chosen for their behavioural relevance. Hausberger [31] described three classes of starling song. Class-I whistles are simple, very loud, and mostly unitary songs. They correspond to four whistle types, namely the inflection (IT), the harmonic (HT), the simple (ST), and the rhythmic (RT) themes, that are found in the repertoires of all male starlings in most populations. These whistles are the basis of song-matching interactions, and they are clearly categorized and recognized by the birds, despite local variations [32]. Only one of them (HT) is occasionally produced by females. Class-II whistles are loud and simple structures composed of one or several notes. They are mostly individual-specific within a colony, but they can be shared by close social partners, both males and females [24], [33]. Finally, class-III songs (also called warbling) are sung in long, complex, and quiet sequences composed of three parts containing motifs that are repeated one to several times with increasing tempo [34], [35]. Most of the motif types are individual-specific, but the second and third parts of a sequence include clicks and high-pitched trills that are found in all male, but not in female sequences.

**Figure 4:**
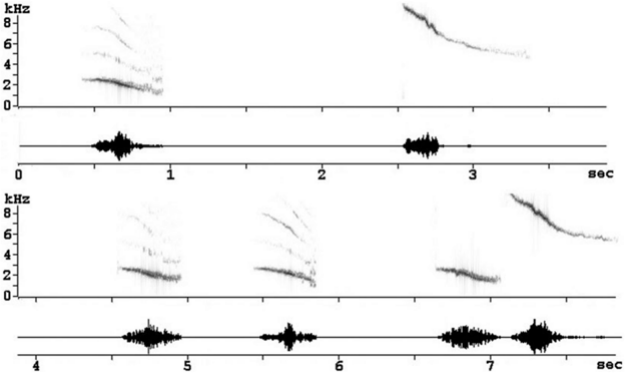
Example of a natural sequence of whistles sung by a wild starling in the field. Sonogram (upper trace) and spectrogram (lower trace) are time aligned [45].

Given that we were mostly interested in the songs' social implication, we decided to put more emphasis on whistles, which are more specifically involved in social exchanges [see], [e.g.], [31,32,33] and not on warbling song, which is involved in mate choice and breeding [34], [35]. Starlings tend to sing successions of whistles separated by 1–8 seconds. Such sequences can include successions of up to 200 whistles, with repetitions of each whistle type in the repertoire (Fig. 4). According to the social context, these successions of whistles may be followed, or not, by a sequence of continuous warbling [32], [36].

Class-I and class-II whistles were chosen for their social relevance: we used each type of class I universal whistles that are usually used in male-male interactions, and unfamiliar, familiar and bird's own exemplars of class II individual-specific whistles [31]. This covered the whole range of starlings' whistle repertoires [37]. Familiar whistles were whistles that had been heard by the birds (adult songs) but that were not present in their own repertoire. The stimuli were broadcast with intervals of 300 ms. This time interval was sufficient to avoid adaptation to stimuli. This method has been used for several decades and no adaptation has been reported in the Field L using this kind of stimulus set [38]. The stimulus set was presented in an anechoic, sound-attenuating chamber through a loudspeaker placed 20 cm in front of the bird's head. The maximum sound pressure at the birds' ears was 60 dB SPL measured by a sound calibrator (LEA S.S.T.4S). The stimulus set was repeated 10 times at each recording site.

### Multi-unit recordings

All neuronal recordings were made during the non-breeding season (autum and winter) in order to avoid possible seasonal influences, as known in other songbirds.

Multiunit recordings were chosen here to characterize neuronal preferences in the field L. This recording method is very stable and allowed us to record activity from a large number of neuronal sites (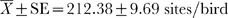). Whereas such recordings do not enable precise evaluation of single cell selectivities, they do give a gross idea of the local neuronal “preferences” [39], [40], [41], [42].

Before the neurophysiological experiments, a stainless steel well was implanted stereotaxically on the bird's skull under halothane anaesthesia (0.4 l/min of carbogene - 95% O2 – 5% CO2 - saturated in halothane - 2bromo-2chloro-1,1,1trifluoroéthane - and 0.6 l/min of carbogene). After implantation, the birds were allowed to rest for 3 days, during which they were kept in cages with conspecifics. During the experiments, the well was used for fixation of the head and as the indifferent electrode.

The electrodes were made by Frederick Haer & Co. (Bowdoinham, USA) and consisted of a tungsten wire insulated by epoxylite, with a fine tip (angle 10–15°). The range of the electrode impedance was 2–4 MΩ. An Amiga 4000 computer was used to record action potentials. A home-made analogue/digital card was used to digitize the recordings (22 kHz, 8 bits), and action potentials were counted with a programmed window discriminator.

The implant was located precisely with reference to the bifurcation of the sagittal sinus: 2.5 mm rostral and 1 mm in the left hemisphere. These values were the coordinates of the centre of the recording plane that was parallel to the sagittal plane. The recording planes were at precisely the same locations for all birds. Recordings were performed at 30 to 40 sites along the path of one electrode penetration. One recording session usually lasted about 3 hours. During recording sessions, the birds were awake and kept in a jacket in order to limit their movements. The recorded plane covered a large part of field L centred on the L2 sub-area described in wild starlings [40]. Penetrations within one recording plane were 200 µm apart. Recordings started, for each penetration, 600 µm below the brain surface, at a site that gave no auditory response, and continued until 4000 µm below the brain surface, where auditory responses were no longer detectable. The recording plane was considered completed when no response was obtained in both outermost penetrations. Twelve penetrations were necessary to complete a recording plane for most animals. The dimensions of the recording plane were 2.4 mm caudo-rostral and 3.6 mm dorso-ventral (8.64 mm^2^ area). After the last recording session, four recording sites were marked by injecting alcian blue to provide orientation points to check the location of the electrode tracks in the forebrain [e.g. 19], [39], [40].

### Data analysis

Experimental data were recorded with a temporal resolution of 0.1 ms. Peri-stimulus time histograms (PSTHs) were calculated, using a temporal resolution of 2 ms, for all the recording sites and all the stimuli. Spontaneous activity was determined from the recording of activity during 100 ms before the beginning of each auditory stimulus. To determine whether there was an activation or an inhibition, the evoked activity was compared to the spontaneous activity using a Student-Fisher t test. We decided that there was activation when p was below 0.01. Since we were trying to determine whether there was a response or not, we were confident in our results using a 0.01 level. However, given the low number of spikes during spontaneous activity (2–3.5 spikes/s), the contrast between spontaneous activity and inhibition was difficult to confirm statistically. We therefore decided to use a p-value of 0.05 for inhibition, which is still a good level [43].

Different measures of responses were made:

- Proportions of responsive sites that differ according to early experience: these proportions are larger in inexperienced animals [19], [21].
- The degree of specialization of the neurons, which was difficult to characterize because of the difficulty to evaluate selectivity properly [44]. We chose an indirect evaluation of neuronal specialization: -1- the proportion of neuronal sites that responded to only one stimulus (specialized sites) and -2- the proportion of sites that responded to all stimuli (generalist sites). This measure proved to be useful in a previous study on developmental plasticity [19].
